# Identification of Epigallocatechin-3- Gallate as an Inhibitor of Phosphoglycerate Mutase 1

**DOI:** 10.3389/fphar.2017.00325

**Published:** 2017-05-30

**Authors:** Xiaoguang Li, Shuai Tang, Qian-Qian Wang, Elaine L.-H. Leung, Hongyue Jin, Yongzhuo Huang, Jia Liu, Meiyu Geng, Min Huang, Shengtao Yuan, Xiao-Jun Yao, Jian Ding

**Affiliations:** ^1^Jiangsu Key Laboratory of Drug Screening, China Pharmaceutical UniversityNanjing, China; ^2^Shanghai Institute of Materia Medica, Chinese Academy of SciencesShanghai, China; ^3^Macau Institute for Applied Research in Medicine and Health, State Key Laboratory of Quality Research in Chinese Medicine, Macau University of Science and TechnologyMacau, China

**Keywords:** phosphoglycerate mutase 1 (PGAM1), inhibitor, cancer metabolism, natural products, anticancer activity

## Abstract

Targeting metabolic enzymes is believed to provide new therapeutic opportunities for cancer therapy. Phosphoglycerate mutase 1 (PGAM1) is a glycolytic enzyme that importantly coordinates glycolysis, pentose phosphate pathway (PPP) flux and serine biosynthesis in cancer cells and hence gains increasing interest of inhibitor discovery. Only few PGAM1 inhibitors have been reported and the molecular potency remains very limited. In an effort to discover new PGAM1 inhibitors, we carried out a biochemical assay-based screen that was focused on natural products derived small molecule compounds. (-)-Epigallocatechin-3-gallate (EGCG), the major natural catechins of green tea extract, was identified as a PGAM1 inhibitor that was tremendously more potent than known PGAM1 inhibitors. Further studies combining molecular docking and site-specific mutagenesis revealed that EGCG inhibited PGAM1 enzymatic activity in a manner independent of substrate competition. EGCG modulated the intracellular level of 2-phosphoglycerate, impaired glycolysis and PPP and inhibited proliferation of cancer cells. This study suggested EGCG as a chemical scaffold for the discovery of potent PGAM1 inhibitors and gained mechanistic insights to understand the previously appreciated anticancer properties of EGCG.

## Introduction

Metabolic reprogramming has been recognized as a hallmark of cancer (Hsu and Sabatini, 2008; Kroemer and Pouyssegur, 2008; Vander Heiden et al., 2009; Ward and Thompson, 2012). Cancer cells are in favor of aerobic glycolysis, also known as ‘Warburg effect,’ which describes a phenomenon that features an increase in aerobic glycolysis and enhanced lactate production even in the presence of oxygen (Warburg, 1956). Aerobic glycolysis confers a significant growth advantage of cancer cells by supplying essential ATP production, generating precursors for biosynthesis, and providing reducing equivalents for antioxidant defense. In this process, glycolytic enzymes play a fundamental role in maintaining the metabolite flux, and in the meanwhile, converging the oncogenic signals to modulate metabolic profile (Mankoff et al., 2007).

Phosphoglycerate mutase 1 (PGAM1) is a mutase that catalyzes the reversible reaction of 3-phosphoglycerate (3-PG) to 2-phosphoglycerate (2-PG) in the glycolytic pathway (Fothergill-Gilmore and Watson, 1989; Hitosugi et al., 2013). A recent work has provided new insights into the function of PGAM1 during aerobic glycolysis, which suggests a role in permitting high level of the pentose phosphate pathway (PPP) flux and biosynthesis via maintaining the intracellular 3-PG at a low level. The upregulated PGAM1 activity in cancer cells, which occurs in multiple types of cancer including breast cancer, hepatocellular carcinoma and colorectal cancer, in turn fulfills the requirement of energy and biomacromolecules of tumor growth. Accordingly, downregulation of PGAM1 expression or inhibition of its metabolic activity leads to attenuated cell proliferation and tumor growth (Durany et al., 1997; Yeh et al., 2008; Ren et al., 2010; Hitosugi et al., 2012).

Recently, there is increasing interest in the discovery of PGAM1 inhibitors to seek potential opportunities in anticancer drug discovery. Two PGAM1 inhibitors, MJE3 (Evans et al., 2007) and PGMI-004A (Hitosugi et al., 2012), have been reported, both of which are currently undergoing early preclinical research. Pharmacological inhibition of PGAM1 by PGMI-004A, a well-validated PGAM1 inhibitor, exhibited evident anticancer properties in mice models carrying human xenograft tumors (Hitosugi et al., 2012). However, the molecular potency of the reported inhibitors are very limited, suggesting the need to explore chemical space. To discover new PGAM1 inhibitors, we focused on natural products that are believed to provide rich chemical resources for drug discovery. This effort discovered (-)-Epigallocatechin-3-gallate (EGCG), the major natural catechins of green tea extract (Yang, 1997; Yang and Wang, 2011), as a potential PGAM1 inhibitor. This study aims to understand how EGCG inhibits the enzymatic activity of PGAM1 and to further prove its potential in modulating cancer metabolism.

## Materials and Methods

### Compounds

A chemical library composed of 800 single purified compounds derived from natural products were purchased from MicroSource Discovery Systems/Topscience (Shanghai, China). EGCG used in the rest experiments in this study was purchased from Selleck (Catalog No. S2250).

### Cell Lines

NCI-H1299 and MDA-MB-231 cells were purchased from American Type Culture Collection (ATCC). PGAM1 stable knockdown sublines were as described previously (Zhang et al., 2016). All cell lines were authenticated and maintained in appropriate culture medium as the suppliers suggested.

### Cell Proliferation Assay

Cells were seeded into a 6-well plate. After attachment, cells were treated with a series concentrations of EGCG or PGMI-004A. The cells were harvested after incubation for 72 h and the proliferation inhibition rate was calculated by cell number counting (Beckman).

### Purification of Recombinant Proteins

BL21 competent cells were transformed by His-tagged pETDuet-1-PGAM1 (wildtype or mutants) or His-tagged pETDuet-1-PHGDH constructs. Cells were collected, resuspended in buffer A (20 mM Tris, 200 mM NaCl, 20 mM imidazole, pH 8.0) and sonicated on ice until complete lysis. His-tagged PGAM1, PGAM1 mutants or PHGDH recombinant proteins in supernatant were purified using Ni-NTA beads (QIAGEN), washed with buffer A, and eluted with buffer B (20 mM Tris, 200 mM NaCl, 250 mM imidazole, pH 8.0). All obtained proteins were stored at -80°C.

### PGAM1 Inhibitors Screening Assay

Recombinant PGAM1 enzymatic activity was measured by a multiple enzymes coupled assay. In brief, assay was conducted in 10 μL buffer (50 mM HEPES, PH 7.5, 10 mM MgCl_2_) containing 5 μL 1 mg/ml recombinant PGAM1 enzyme, 2.5 μL test compound, 2.5 μL 1 mM ADP, 1 mM 3-PG, 0.5 units/ml enolase (Sigma–Aldrich) and 0.5 units/mL recombinant pyruvate kinase M1 (Sigma–Aldrich). The production of ATP was measured by Kinase-Glo^®^ Max Luminescent Kinase Assay (Promega).

### IDH Enzymatic Assay

Assays were conducted in 10 μL buffer [150 mM NaCl, 20 mM Tris pH7.5, 10 mM MgCl_2_, 0.05% (w/v) bovine serum albumin] containing 2.5 μL of test compound, 5 μL enzyme solution (10 ng/μL mutant IDH, Cayman Chemical) and 2.5 μL substrate solution (1 mM α-KG, 4 μM NADPH) added into a 384-well plate followed by incubation at room temperature for 60 min. After incubation, NADPH remaining amount was measured by adding 5 μL buffer containing 5 μM resazurin and 0.01 unit diaphorase and incubated at room temperature for 10 min. The plate was read using EnVision (Perkin Elmer) at Ex 540 Em 590 nm.

### PHGDH Enzymatic Assay

Assays were conducted in 10 μL buffer (200 mM Tris-HCl pH8.1, 400 mM KCl, 2 mM GSH, and 10 mM EDTA) containing recombinant PHGDH, 0.6 mM NAD, 1 mM 3-PG. The NADH product can be measured by diaphorase/rezasurin system.

### Surface Plasmon Resonance Spectroscopy

Surface plasmon resonance (SPR) assay was carried out with a ProteOn XPR36 instrument (Bio-Rad). PGAM1 was coupled to the surface of a GLH sensor chip according to the manufacturer’s instructions. To measure the binding affinity to PGAM1, a series concentrations of EGCG or PGMI-004A were run over the surface of the chip at 25°C with a constant flow rate of 30 μL/min in running buffer (10 mM Phosphate, 150 mM NaCl, pH 7.4 and 0.005% Tween20 [v/v]). Resonance units (RU) changes represent the binding condition. The dissociation constant (*K*_d_) was calculated using the ProteOn Manager 3.1 software program by fitting to a 1:1 Langmuir binding model.

### Molecular Docking Calculation

The 3-dimensional structure of citrate-PGAM complex was obtained from the Protein Data Bank (PDB ID: 1YFK). The protein was prepared with the Protein Preparation Wizard. The structures of PGMI-004A and EGCG were downloaded from ZINC database, and preprocessed by LigPrep under the OPLS-2005 force field (Jorgensen et al., 1996). PGMI-004A or EGCG was docked into the binding site of PGAM protein using the Glide module (Friesner et al., 2006) with the standard precision score. The docking grid box of the inhibitors on PGAM1 was defined according to the citrate in the original complex (PDB ID: 1YFK). For each system, the pose with the best score was chosen for further analysis. All the molecular docking calculation was carried out with the Schrödinger 2015 software.

### Solubility and Permeability Measurement

The solubility of compounds was measured by Nephelometry using the NEPHELOstar plus apparatus (BMG Lab Technologies). Caco-2 monolayer was cultivated on permeable support. In details, after 21-day culture, the integrity of the cell monolayer of every well was verified by measuring the transepithelial electrical resistance (TEER). The diluted solution was added to corresponding well and the plate was incubated at 37°C for 1.5 h. 100 μL samples were collected from both apical and basolateral sides at the initial and end time points and the sample concentrations were measured for the evaluation of mass balance.

### Liposome EGCG Assay

Liposomes were prepared by the thin film hydration method. Briefly, cholesterol, DOTAP, DSPE-PEG2000 (molar ratio 23: 69: 8) were dissolved in methanol in a pear-shaped flask. After methanol was removed by rotary evaporation, and the film was formed and further dried under vacuum overnight. Specifically, EGCG was loaded in the liposomes by adding to organic solution. The film was hydrated by 5% glucose solution at 42°C for 5 min followed by sonication (50 W). The liposomes were extruded through 400 and 100 nm polycarbonate filters sequentially.

### Intracellular NADPH/NADP^+^ Measurement

A NADPH/NADP^+^ kit (BioAssay Systems) was used to measure cellular NADPH/NADP^+^ ratios. Cells were harvested after 24-h treatment, washed with PBS, and then lysed with 200 μL of NADP^+^ (or NADPH) extraction buffer. Heat extraction was allowed to proceed for 5 min at 60°C before adding 20 μL assay buffer and 200 μL the counter NADPH (or NADP^+^) extraction buffer to neutralize the extracts. The supernatants were reacted with working buffer according to the manufacturer’s protocol. The absorbance was measured at 570 nm.

### Intracellular 2-PG Measurement

A 2-Phosphoglycerate Colorimetric/Fluorometric Assay kit (BioVision) was used to measure cellular 2-PG level. Cells were collected after exposure to compounds for 24 h, washed with PBS, and lysed with ice-cold 2-PG assay buffer. The extracts were spun down to remove cell debris and the supernatant was reacted with working buffer according to the manufacturer’s instructions. The samples were measured by using EnVision^®^ at Ex 535 Em 587 nm.

### Intracellular Lactate Measurement

A Lactate Colorimetric/Fluorometric Assay Kit (Biovision) was used to measure the intracellular lactate levels. After exposure to compounds for 24 h, cells were lysed and centrifuged to collect the cell supernatant. The supernatant of cell lysates was mixed with the assay solution. The absorbance was measured at 570 nm.

### Extracellular Acidification Rate Analysis

Cells were seeded into Seahorse XF96 cell culture plates and were treated with indicated compounds for 24 h. Each XF96 assay well was equipped with a disposable sensor cartridge coupled to fiber-optic waveguides. The extracellular acidification rate was measured using a Seahorse XF96 analyzer (Seahorse Bioscience).

## Results

### Discovery of EGCG as a PGAM1 Inhibitor *In Vitro*

**FIGURE 1:**
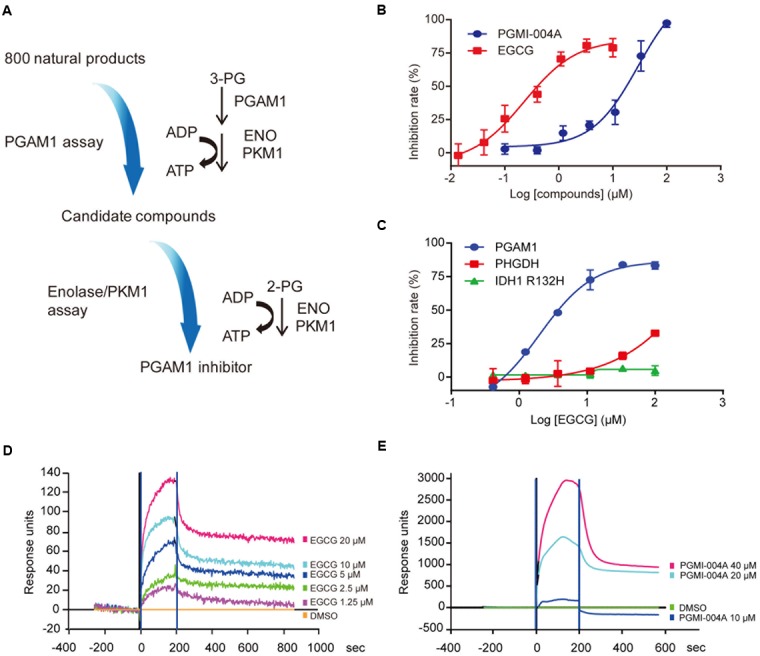
(-)-Epigallocatechin-3-gallate (EGCG) inhibits Phosphoglycerate mutase 1 (PGAM1) catalytic activity *in vitro*. **(A)** Flow chart of the screening strategies to identify PGAM1 inhibitors *in vitro*. **(B)** Inhibition of PGAM1 catalytic activity by EGCG *in vitro*. PGMI-004A was used as a control. **(C)** Impact of EGCG on the activity of IDH1 R132H or PHGDH; **(D,E)** SPR analysis of molecular interaction between EGCG **(D)** or PGMI-004A **(E)** with PGAM1. Various concentrations of EGCG or PGMI-004A were injected into PGAM1-immobilized chip and the *K*_d_-value was calculated using the ProteOn Manager 3.1 software program by fitting to a 1:1 Langmuir binding model.

To identify new PGAM1 inhibitors, we utilized a coupled PGAM1 and pyruvate kinase isozymes M1 (PKM1)/enolase biochemical assay followed by a counter screen to exclude compounds with activity toward PKM1 or enolase. A reported PGAM1 inhibitor, PGMI-004A, was used as a positive control (**Figure 1A**). Using these biochemical assays, we screened a chemical library composed of 800 purified compounds derived from natural products. EGCG was identified as a potential PGAM1 inhibitor. We further showed that EGCG inhibited PGAM1 in a dose-dependent manner, with an IC_50_ of 0.49 ± 0.17 μM, over 30 times more potent than that of PGMI-004A (IC_50_ = 18.62 ± 2.24 μM, **Figure 1B**). In the meanwhile, EGCG did not inhibit other metabolic enzymes like phosphoglycerate dehydrogenase (PHGDH) or mutant isocitrate dehydrogenase 1 (IDH1) at a concentration up to 100 μM, suggesting the specific inhibition of EGCG toward PGAM1 (**Figure 1C**).

To prove whether EGCG directly binds to PGAM1 to inhibit its enzymatic activity, we used the SPR assay to examine the molecular interaction between EGCG and PGAM1. Recombinant human PGAM1 was immobilized on the chip surface and EGCG was provided in the fluidic phase at different concentrations to determine its affinity to PGAM1. PGMI-004A was tested in parallel. Both compounds directly bound to PGAM1. The dissociation constant (*K*_d_-value) of EGCG was 0.78 μM whereas that of PGMI-004A was 54.8 μM (**Figures 1D,E**), consistent with their respective potency in the biochemical enzymatic assay (**Figure 1C**). The results showed that EGCG exhibited a high binding affinity to PGAM1, which may explain its potency toward PGAM1 *in vitro*.

### Molecular Modeling of EGCG and PGAM1 Interaction

The apparent advantage of EGCG in its binding affinity to PGAM1 intrigued us to understand its binding mode to PGAM1. To address this question, we took advantage of previously resolved crystal structure of PGAM1 (Wang et al., 2005) and used molecular docking approach to compare the binding modes of EGCG and PGMI-004A on the surface of PGAM1. The Glide SP docking score for PGMI-004A and EGCG systems were -4.68 and -6.55 kcal/mol, respectively, which agreed with their respective molecular potency in the enzymatic study.

**FIGURE 2:**
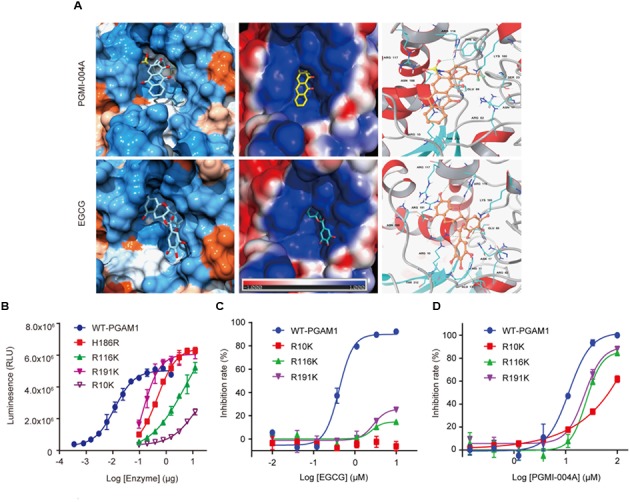
Binding mode of EGCG to PGAM1. **(A)** Molecular docking of the binding mode of EGCG and PGMI-004A with PGAM1; **(B)** Enzymatic activity of PGAM1 mutants. **(C)** EGCG inhibition against PGAM1 mutants; **(D)** PGMI-004A inhibition against PGAM1 mutants.

According to the docking results, most of the residues interacting with the PGMI-004A and EGCG were basic. More H-bonds were formed with EGCG relative to that with PGMI-004A. PGMI-004A formed three H-bonds with R116 and R10 while EGCG formed eight H-bonds with R10, R116, R191, E13, E89, and N209. The stronger H-bond interactions of the latter was mainly from the phenolic hydroxyl groups of EGCG. From the view of structure, the molecular volume of EGCG was notably larger than PGMI-004A. However, due to the high flexibility, EGCG was able to bind well with PGAM1 (**Figure 2A**). More hydroxyl groups and structural flexibility together may explain the highly improved molecular potency EGCG toward PGAM1.

To further confirm the aforementioned binding mode of EGCG, we introduced point mutations to the key residues that were identified to interact with EGCG in the binding pocket. Measurement of the enzymatic activity of the individual mutant showed that R191K and R116K mutants maintained some catalytic activity whereas R10K mutant largely lost the catalytic capacity of PGAM1, suggesting that these residues were essentially required for the enzymatic activity of PGAM1. H186R was a well-validated catalytically dead mutant of PGAM1 (Zhang et al., 2016; Qu et al., 2017), which was used as a control (**Figure 2B**). We next measured the impact of these mutations on the activity of EGCG. Importantly, EGCG lost its inhibitory activity toward the R10K, R116K, and R191K mutants compared to the wild-type PGAM1 (**Figure 2C**). In parallel, point mutations to these residues only slightly affect the activity of PGMI-004A (**Figure 2D**). These data demonstrated that EGCG inhibited PGAM1 activity via interacting with the R10, R116, and R191 residues.

### EGCG Inhibits PGAM1 Independent of 3-PG Competition

**FIGURE 3:**
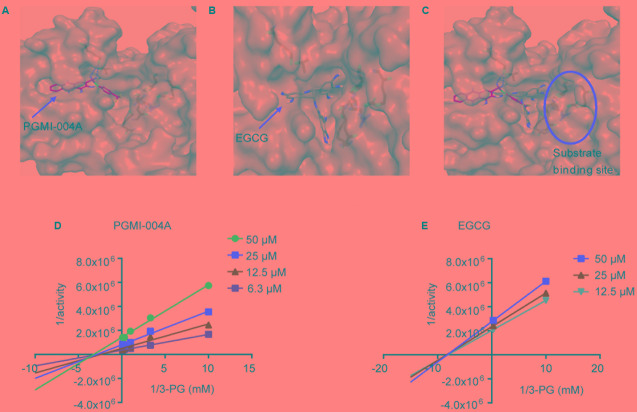
(-)-Epigallocatechin-3-gallate inhibits PGAM1 independent of substrate competition. **(A)** The binding site of PGMI-004A (red arrow) in PGAM1 (gray surface). **(B)** The binding site of EGCG (red arrow) in PGAM1. **(C)** The substrate 3-PG binding site (red circle) in PGAM1. **(D,E)** Substrate 3-PG competitive analysis determined by Lineweaver-Burk plots.

In addition to providing a better understanding of how EGCG interacts with PGAM1, it was also noted that the binding site of EGCG suggested by the molecular docking was adjacent to the reported 3-PG binding site (**Figures 3A–C**). We asked whether EGCG exhibited an inhibitory mechanism via substrate competition with 3-PG. To test this possibility, the Lineweaver-Burk plot analysis was used to investigate the competitiveness between EGCG and 3-PG. The analysis revealed that EGCG exhibited a non-competitive pattern with its substrate 3-PG, similar to PGMI-004A (**Figures 3D,E**).

### EGCG Inhibits Glycolysis and Proliferation of Cancer Cells

**FIGURE 4:**
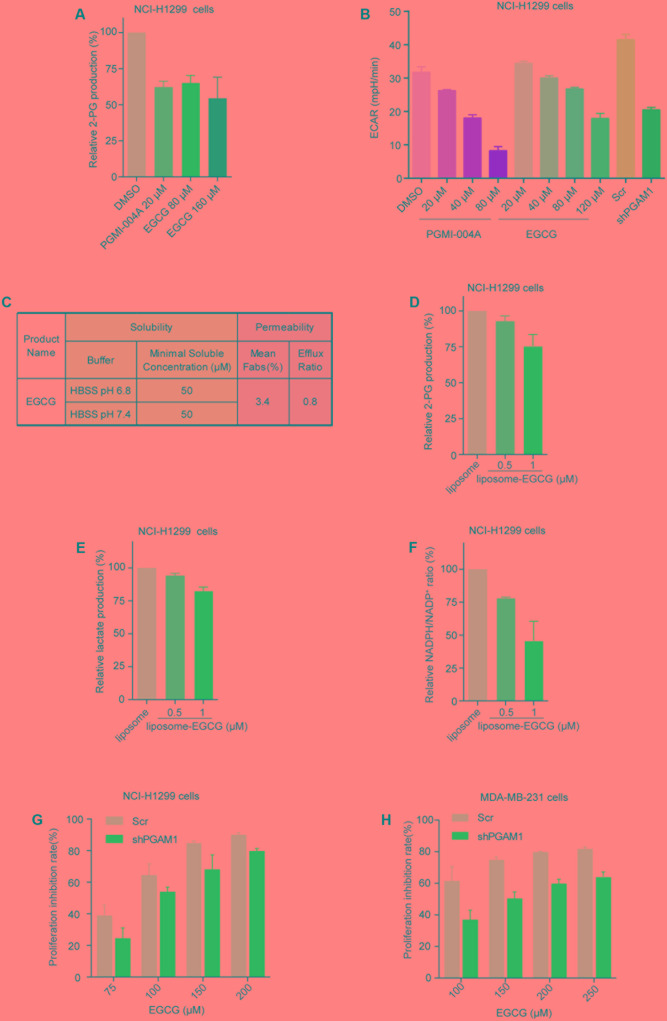
(-)-Epigallocatechin-3-gallate inhibits PGAM1 activity in cancer cells. **(A)** Impact of EGCG on intracellular 2-PG production in cancer cells; NCI-H1299 cells were treated with indicated compounds for 24 h. **(B)** Impact of EGCG on lactate production. NCI-H1299 cells were treated with indicated compounds for 24 h and extracellular lactate release was determined by Seahorse XF 96. ECAR represent extracellular H^+^ concentration; PGAM1 stable knockdown cells (shPGAM1) and scramble (Scr) control cells were used as a control. **(C)** Solubility and permeability of EGCG. **(D–F)** Cells were treated with liposome-EGCG at indicated concentrations for 24 h and intracellular 2-PG concentration **(D)**, extracellular lactate production **(E)** and NADPH/NADP^+^ ratio **(F)** were determined. **(G,H)** PGAM1 dependent inhibition of cancer cell growth by EGCG. PGAM1 stable knockdown (shPGAM1) or scramble (Scr) control cells were treated with EGCG at indicated concentrations for 72 h and cell growth was determined by counting viable cells.

PGAM1 is known to modulate glycolysis, PPP and biosynthesis via its activity in converting 3-PG to 2-PG (Hitosugi et al., 2012, 2013). We next proceeded to examining whether the molecular potency of EGCG could lead to effective inhibition of glycolysis and in turn growth of cancer cells. We firstly assessed the impact of EGCG on the intracellular generation of 2-PG (Hitosugi et al., 2012; Zhang et al., 2016). NCI-H1299, a human non-small cell lung carcinoma cell line, was treated with EGCG for 24 h and intracellular concentration of 2-PG was measured. EGCG decreased intracellular 2-PG level in a dose-dependent manner. Unexpectedly, apparent inhibition was only observed when EGCG concentration was increased to 80 μM, comparable to 20 μM of PGMI-004A (**Figure 4A**). This result was consistent with the effect of EGCG on extracellular acidification rate (ECAR), which indicated the capacity of glycolysis in cells (**Figure 4B**). We reasoned that the concentration gap between molecular and cellular potency of EGCG likely resulted from its polyphenol structure caused low membrane permeability. We hence measured the permeability and solubility of EGCG, and confirmed the extremely low membrane permeability of EGCG (**Figure 4C**). To clear this hurdle, we chose to utilize liposome as a carrier to facilitate intracellular entry of EGCG. Indeed, with the assistance of liposome, EGCG was able to inhibit intracellular 2-PG production at a concentration as low as 1 μM, consistent with its molecular potency (**Figure 4D**). Similar results were observed in the lactate production and the NADPH/NADP^+^ ratio (**Figures 4E,F**). These results suggested that EGCG could attenuate cancer cell glycolysis and PPP by inhibiting the activity of PGAM1.

Anticancer properties of EGCG have long been observed, which are attributed its broad impacts on a spectrum of molecules, including epidermal growth factor receptor (Ma et al., 2014), mitogen activated protein kinase (Gao et al., 2013), telomerase (Sadava et al., 2007; Berletch et al., 2008), polo-like kinase 1(Shan et al., 2015), and the proteasome (Kazi et al., 2004; Lam et al., 2004). To prove whether PGAM1 inhibition contributed to the anticancer activity of EGCG, we compared its anticancer activity in a pair of cancer cells with or without PGAM1 depletion. We constructed PGAM1 stable knockdown NCI-H1299 cells and tested the cell sensitivity to EGCG. We observed that EGCG dose-dependently inhibited cell growth while stable knockdown of PGAM1 partially rescued it-caused growth inhibition, suggesting a PGAM1 dependent cellular activity (**Figure 4G**). Similar results were obtained in a breast cancer cell line MDA-MB-231 cells (**Figure 4H**). These results collectively suggested that EGCG exhibited an inhibitory activity toward PGAM1, leading to attenuated glycolysis, PPP and in turn cell growth.

## Discussion

Targeting aerobic glycolysis of cancer cells has been believed to open the new therapeutic opportunities for cancer therapy (Shaw, 2006; Porporato et al., 2011; Ganapathy-Kanniappan and Geschwind, 2013; Zhao et al., 2013; Hay, 2016; Li and Zhang, 2016). There is increasing interest in the field of anticancer drug discovery in exploring metabolic enzymes, yet only a few inhibitors have been validated in their anticancer promise. Among the glycolytic enzymes, PGAM1 sits at a unique position in coordinating both aerobic glycolysis pathway and anabolic biosynthesis, making it an Achilles heel in glucose metabolism regulation. Of note, our recent studies have also revealed that PGAM1 inhibition inactivated homologous recombination repair and exhibited synthetic lethality with PARP inhibitors in cancer cells, which further inspired the interest to develop PGAM1 inhibitors for the treatment of cancer (Qu et al., 2017).

Currently, there lacks clear strategy in the rational design of new PGAM1 inhibitors or in optimizing the activity of reported PGAM1 inhibitors, as their molecular mechanism remain unclear. To explore new chemical scaffolds could gain in-depth insights for this target and may bring new opportunities for drug discovery. In this study, we chose to seek opportunities from natural products, which represent a rich source of compounds with enormous structural diversity and have been extensively explored in the field of drug discovery with remarkable successes. This effort led to the discovery of a natural chemical EGCG, a major polyphenol derived from green tea, as a potent inhibitor of PGAM1. EGCG exhibits a molecular potency over 30 times more potent than the known PGAM1 inhibitors, mainly PGMI-004A, which may provide a new chemical scaffold for future medicinal chemistry studies.

It remains unclear how EGCG inhibits the catalytic activity of PGAM1. Our data, from both molecular docking and biochemistry assay, together excluded a possibility of substrate competition, which is also the case for PGMI-004A. It was noted that the arginine residues R10, R116, and R191 that are interacting with PGAM1, are essential for the enzymatic activity of PGAM1, as point mutation to either of these residues led to great enzymatic activity loss of PGAM1. EGCG binding site was very close to 3-PG binding site while they did not compete with each other. We hence speculated that EGCG binding may possibly result in conformational change in the surroundings residues, which affects the substrate binding and in turn disrupts the enzymatic activity of PGAM1, though more evidence are still required for proving this possibility.

It has been reported that EGCG also inhibits the expression of hexokinase 2 (HK2), one of the rate limiting enzyme of the aerobic glycolysis, partially through the down-regulation of Akt signaling pathway in human tongue carcinoma (Gao et al., 2015). Our study showed that EGCG directly inhibited PGAM1 activity and decreased the intracellular level of 2-PG. These together suggested that EGCG may attenuate the glycolysis rate via multiple mechanisms. In line with these insights, depletion of PGAM1 in NCI-H1299 and MDA-MB-231 cells only partially rescue the anti-tumor effects of EGCG. Moreover, PGAM1 inhibitory effect by EGCG revealed by this study also expanded its impact to PPP flux, as showed by the decreased NADPH/NADP^+^ ratio, and presumably the biosynthesis.

Indeed, the anticancer activity of EGCG have long been attributed to a spectrum of targets like epidermal growth factor receptor (Ma et al., 2014), mitogen activated protein kinase (Gao et al., 2013), telomerase (Sadava et al., 2007; Berletch et al., 2008), polo-like kinase 1(Shan et al., 2015) and the proteasome (Kazi et al., 2004; Lam et al., 2004). While most of these studies were lack of biochemistry dataset, all the evidence consistently suggested its cellular potency up to hundreds of micromoles. In these study, by comparing biochemistry and cellular potency, we were able to show that the low membrane permeability was the major cause of the largely compromised cellular activity of EGCG. As a polar molecule, the phenolic hydroxyl groups made EGCG impermeable to the cell membrane, which resulted in the inhibition of EGCG at a very high concentration in cells. The liposome encapsuled EGCG facilitated its penetration across the cell membrane and yielded a significantly improved cellular potency. 1 μM of EGCG could significantly reduce the intracellular generation of 2-PG. These insights may suggest improved membrane penetration as the direction for future efforts in further optimizing the cellular activity of EGCG.

In summary, the enzymatic inhibition of PGAM1 by EGCG brought us new biochemical and pragmatic insights into targeting aerobic glycolysis for cancer therapy. EGCG derivatives with better potencies could be examined in addition to improved methods of delivery. Further chemical search for new chemical scaffolds remains an important aspect for future directions.

## Author Contributions

JD, MG, and SY provided supervision. MH and X-JY conceived the project, provided supervision and wrote the manuscript. XL, ST, and Q-QW performed the research and analyzed the data. HJ and YH assisted liposome preparation. JL assisted measurement of permeability. EL provided the library and assisted screening studies.

## Conflict of Interest Statement

The authors declare that the research was conducted in the absence of any commercial or financial relationships that could be construed as a potential conflict of interest.
